# Measuring adhesion on rough surfaces using atomic force microscopy with a liquid probe

**DOI:** 10.3762/bjnano.8.84

**Published:** 2017-04-10

**Authors:** Juan V Escobar, Cristina Garza, Rolando Castillo

**Affiliations:** 1 Instituto de Física, Universidad Nacional Autónoma de México; P. O. Box 20-364, DF, México, 01000, Mexico

**Keywords:** atomic force microscopy, force of adhesion, functionalized-tip cantilevers, liquid probe

## Abstract

We present a procedure to perform and interpret pull-off force measurements during the jump-off-contact process between a liquid drop and rough surfaces using a conventional atomic force microscope. In this method, a micrometric liquid mercury drop is attached to an AFM tipless cantilever to measure the force required to pull this drop off a rough surface. We test the method with two surfaces: a square array of nanometer-sized peaks commonly used for the determination of AFM tip sharpness and a multi-scaled rough diamond surface containing sub-micrometer protrusions. Measurements are carried out in a nitrogen atmosphere to avoid water capillary interactions. We obtain information about the average force of adhesion between a single peak or protrusion and the liquid drop. This procedure could provide useful microscopic information to improve our understanding of wetting phenomena on rough surfaces.

## Introduction

After the introduction of the atomic force microscope (AFM), it was clear that force-vs-distance curves could be measured using this new instrument and that several fields related to adhesion [1] could be promoted in new directions. One of them was the replacement of the tip with a colloidal particle. Ducker et al. [2–3] and Butt [4] were the first ones in using the newly termed “colloidal-probe technique” [5], which became a standard and powerful tool for the study of surface forces. In particular, this colloidal-probe technique has been useful to characterize the work of adhesion between two solid surfaces (force of adhesion per unit area) that governs contact stresses and strongly influences friction. In such method, the force, *F*_adh_, required to separate a tip from a flat solid sample is measured. Subsequently, a single-asperity continuum contact mechanics model is used to extract the work of adhesion. It is usually assumed that the process takes place in a regime of small strains, that the materials are homogeneous, isotropic, linearly elastic, and that the tips are perfectly smooth. On this last point, previous studies [6–8] have demonstrated the high sensitivity of adhesion to interfacial roughness showing a drop in *F*_adh_ of more than an order of magnitude with increasing roughness, down to the atomic limit [9].

In this paper, we present a procedure to measure the force of adhesion between a liquid drop and rough surfaces, in which a micrometric liquid drop replaces the colloidal particle on a tipless cantilever. The force required to pull the drop off a surface is measured in a nitrogen atmosphere to avoid water capillary interactions. The liquid probe is close to what would be expected to be a smooth probe down to the atomic level. Recently, we reported an instrument and a procedure to measure forces between a liquid drop and flat surfaces when the adhesion is too large to be measured with an AFM [10]. Indeed, in general, adhesion between liquids and solids can be high. However, interactions between a liquid probe and supersolvophobic or highly patterned surfaces are in the appropriate range of AFM force measurements, as we will show below. The novel procedure we use to this end, could be used to provide untapped information about the fundamental understanding of wetting [11–12], and also for practical applications regarding supersolvophobic surfaces [13], in self-cleaning – because liquid-repellency is correlated with a low adhesion force –, drag reduction [14], fog harvesting [15], and to understand adhesive interactions between imaging materials, which are crucial in print-engine design, and print-process development in the printing industry [16]. We point out that the results we present in this work constitute a proof of principle in which some issues could be improved, mainly related to the method to obtain more reliable spring constants [17] to get quantitative and comparable measurements.

To our knowledge, only a few reports study the case of drops attached to AFM cantilevers [18–21]. In all cases, the authors used an AFM to measure the interaction between oil droplets inside a water solution to probe the effect of additives adsorbed on the droplets. On the other hand, relevant works regarding the connection between wetting and adhesion can be mentioned. Samuel et al. [22] found that when a water drop is retracting from a solid surface, the pull-off force correlates well with the receding contact angle. The pull-off force decreases monotonically as the receding contact angle increases. An important phenomenon that also needs to be taken into account is that a drop attached to two flat surfaces can form a liquid bridge that can be compressed and stretched [23–24]; Chen et al. [25] have presented models of the effect of contact angle hysteresis on this phenomenon. Nevertheless, when adhesion is high, the water drop may break during pull-off and results in a small residual water droplet on the surface.

In the present study, we choose mercury as the liquid because it presents many advantages. Hg possesses a very high surface tension and negligible evaporation, plus it is relatively easy to attach to a tipless cantilever. Also, the drop does not break during a pull-off process, and its solvophobic behavior with specific surfaces is known [26]. As rough surfaces, we use a patterned surface composed of a regular array of sharp (nanometer-sized) silicon peaks, and a Hg-phobic multi-scaled diamond surface with heterogeneous location distribution. The topological differences between the two surfaces are reflected in the force of adhesion results we obtain, suggesting it is possible to implement this method as a tool to characterize the interaction between liquids and rough surfaces.

This article is organized as follows: In the Experimental section, materials, experimental procedures and setup are described. In the Results and Discussion section, we first validate our method for determining the pull-off force by measuring the force of adhesion between a commercial Si_3_N_4_ AFM tip and a mica surface. Then, we present our results as well as a discussion of the measured force of adhesion between three pairs of contacting bodies: a) An array of sharp silicon peaks and a mercury drop probe. For comparison, we also present results of force measurements between b) the aforementioned surface composed of sharp silicon peaks and a solid hollow silica sphere of similar dimensions as the mercury drop. As a final pair, we measure the interaction between c) a mercury drop and a Hg-phobic multi-scaled rough diamond surface. Finally, in the last section we present the conclusions.

## Experimental

The measuring principle we use is similar to that employed for measuring forces in the colloidal probe technique [4–5]. The surface under study is moved up and down by applying a voltage to a piezoelectric translator while recording the cantilever deflection. The deflection of the cantilever is measured with the optical beam deflection technique. As the cantilever bends, the reflected light beam spot moves on the detector when the probe and the surface interact. The force of the bent cantilever is directly translated into the signal of the detector in volts and is plotted versus the position of the piezo that moves the surface upwards. To obtain a force-vs-distance curve, the detector signal and the piezo position have to be converted into force and distance. The conversion factor needed to calculate the cantilever deflection from the detector signal is obtained from a linear fit to the constant compliance region of the piezo. The force acting on the cantilever is obtained by multiplying its deflection by the spring constant of the cantilever. In the standard AFM technique, the tip apex typically has a radius of 5–50 nm, whereas the radii of colloidal probes are in the range of 1–100 μm, resulting in much higher adhesion forces.

**Mercury:** Double distilled mercury was first passed through a small orifice on a filter paper and then dropped three times through a column of 30% nitric acid. The clean mercury was washed with distilled water and dried with filter paper. Mercury is kept under chloroform. Mercury is a hazardous liquid that can result in severe body damage if proper care is not taken. Therefore, it was handled in small quantities and kept in capped bottles at all times. All mercury cleaning and handling was performed inside a fume hood following the appropriate safety considerations [27].

**Figure 1:**
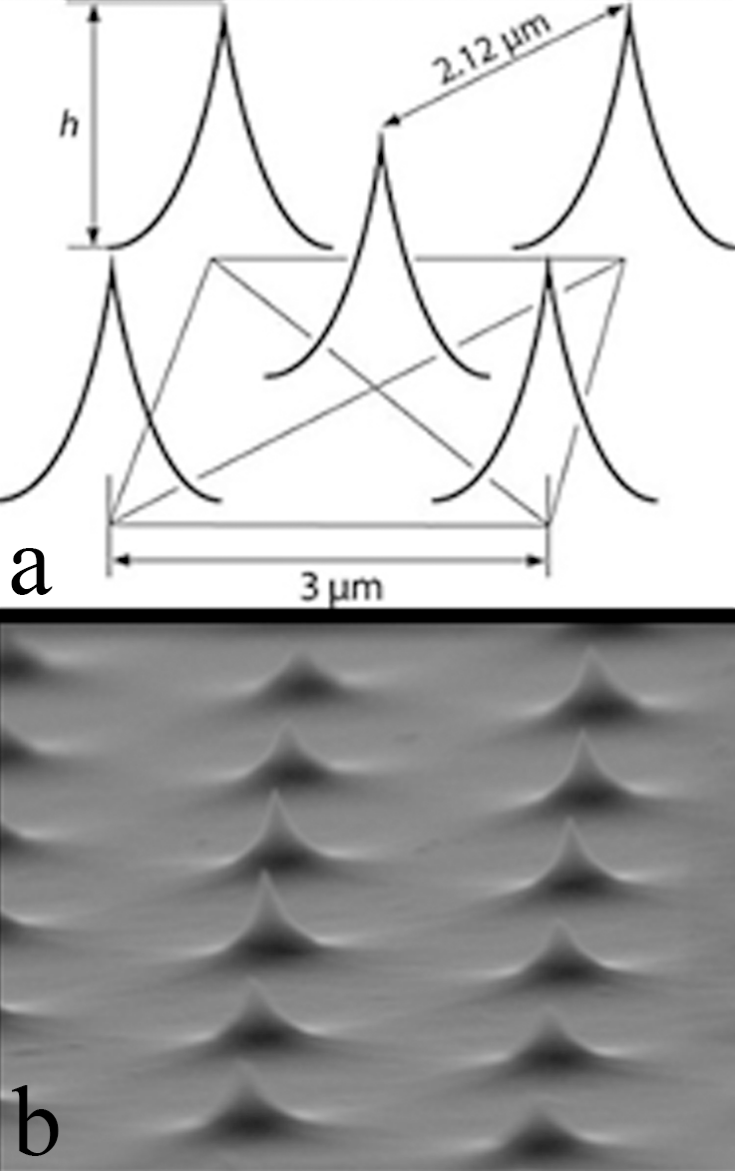
Test grating. a) Lattice features given by the manufacturer. b) Scanning electron microscopy image of the array of sharp silicon peaks of the actual test grating (TGT1) used in our experiments obtained with a JSM7800-LV microscope.

**Surfaces:** Two rough surfaces were used to measure the adhesion force: 1) An array of silicon structures with nanometer-scale peaks (test grating TGT1 from NT-MDT Co., Russia; Figure 1) normally used for determining the radius of curvature of the AFM tip. A macroscopic mercury drop wets this surface following the Cassie–Baxter model [13] (θ_c_ ≈ 150°). The grating consists of a high-resistivity silicon(100) monocrystalline wafer topped with an array of sharp silicon peaks forming a 2D face-centered rectangular lattice whose primitive translation vectors have equal magnitudes of 3 ± 0.01 μm (see Figure 1a). The manufacturer gives nominal parameters for the peaks: peak angle 50 ± 10°, peak radius 10 nm, and peak height 0.3–0.7 μm. These variations in height can be observed in Figure 1b; this piece of information will be important for the analysis of the results below.

**Figure 2:**
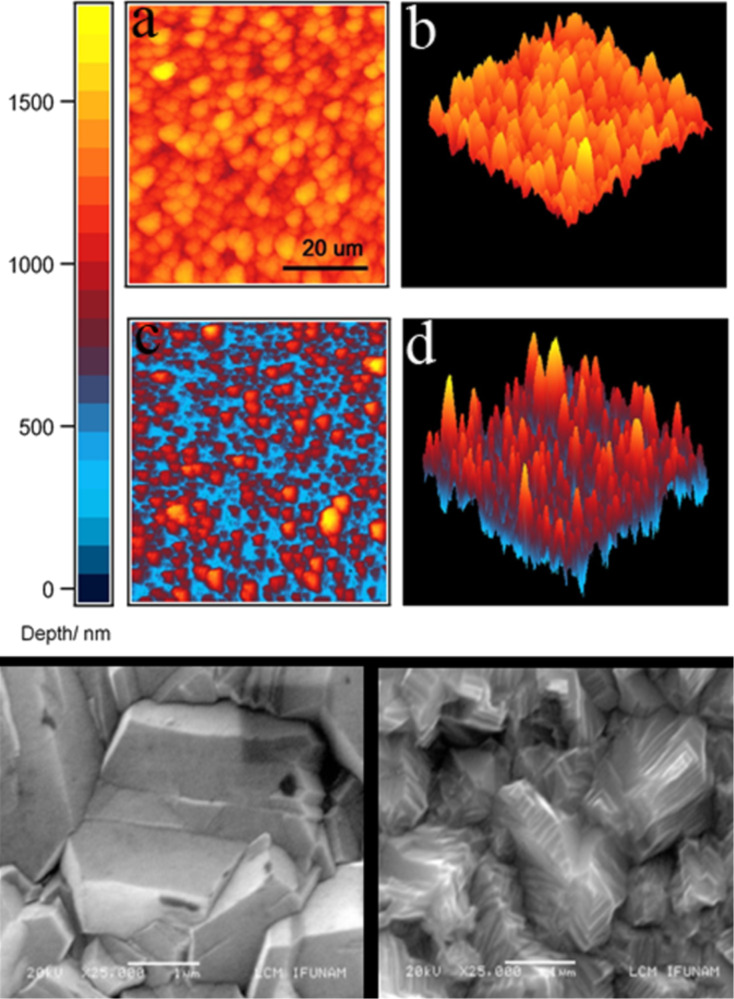
Top panel: AFM profiles of a 60 × 60 μm^2^ portion of the microcrystalline diamond film: (a,b) before and (c,d) after oxidation; reproduced with permission from [26], copyright 2013 Elsevier. Bottom grayscale panels: corresponding SEM images (scale bar = 1 μm) of a typical microcrystal from the diamond film before oxidation (top image) and after oxidation, when a multi-scaled rough surface is obtained (bottom image).

**Figure 3:**
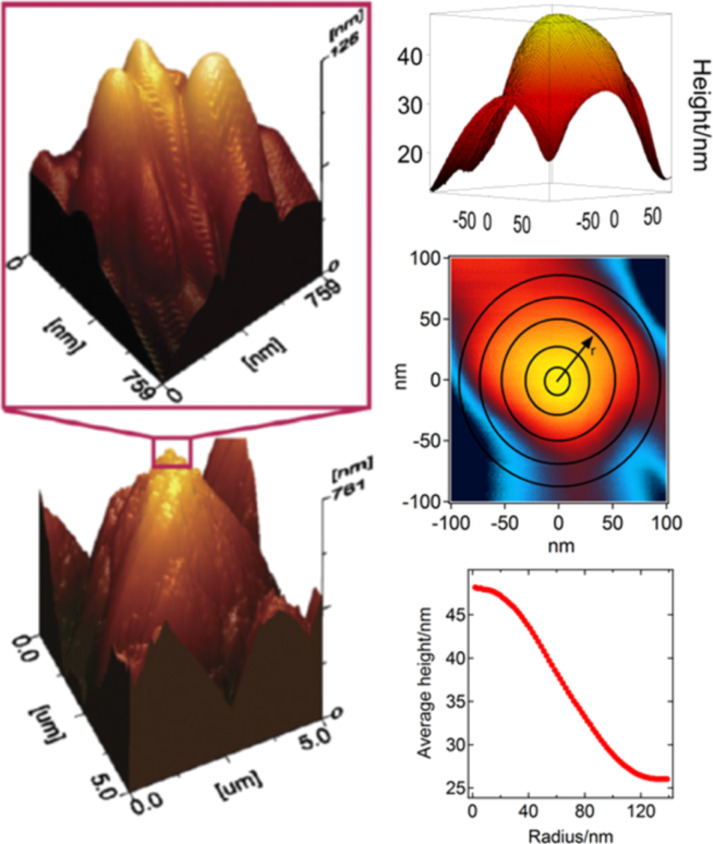
Left: Sub-micrometer protrusions on the pyramids of the multi-scaled rough diamond surface; reproduced with permission from [26], copyright 2013 Elsevier. Right: (top, middle) Typical sub-micrometer protrusion topography obtained with AFM, with the average height as a function of the peak radial distance in the lower right panel.

2) A Hg-phobic multi-scaled rough diamond surface. We modified the roughness of a 7 μm thick boron-doped microcrystalline diamond film deposited on a silicon substrate (SP3 Corporation, USA) via thermal oxidation [26]. The original microcrystalline film has a density of 0.1 microcrystals per square micrometer, with an average area of 10 μm^2^ per crystal, but as opposed to the AFM grating (Figure 1), the crystals are randomly distributed on the surface. This microcrystalline diamond film was heated at 850 °C for 10 min inside a tubular furnace that had its ends open to the atmosphere. This process thermally oxidizes the diamond crystals and removes layers of diamond as carbon monoxide. This surface has been thoroughly characterized by AFM, SEM, and XPS in [26]. Figure 2 presents a survey of the original and the thermally oxidized films. Mayan-pyramid-like structures are formed after thermal oxidizing the diamond films, as observed with SEM, whose tops have linear dimensions of the order of 200 nm. Nevertheless, observation of the pyramid surfaces with AFM reveals that the tops are decorated with ca. 100 nm high protrusions. Figure 3 presents the sub-micrometer protrusions decorating the pyramids of the multi-scaled rough surfaces, and their typical average height as a function of the radial distance measured from the center of the peak. Thus, this oxidized surface is rough on both the nano- and the micro-scales, a necessary condition to achieve supersolvophobic states for mercury drops [26]. As a result, mercury drops lying on the surface would be in contact with a much smaller effective area.

**Mercury-drop probes:** A drop of mercury is taken from the inside of clean mercury using a syringe. The drop is skimmed off with a Teflon device to discard any possible remaining or newly formed oxide. A small amount of mercury is sucked out again from the inside of the remnant mercury, and it then is squeezed between two freshly cleaved mica surfaces to spread the liquid on one of the surfaces. This procedure allows us to select a droplet of 10–30 μm in diameter, which is then attached to an AFM tipless cantilever with the AFM approach system. To this end, the cantilever is previously covered with a sticky adhesive as explained below.

**Figure 4:**
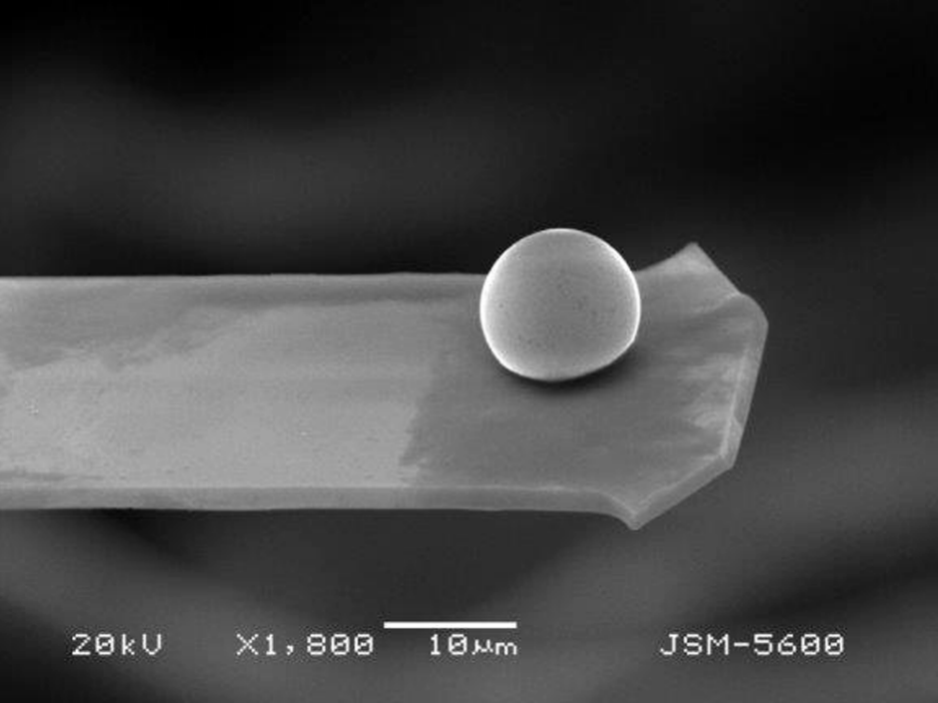
Scanning electron microscopy image of a mercury drop attached to a tipless cantilever obtained with a JSM5600-LVmicroscope.

**Tip coating with a sticky adhesive:** The sticky glue portion of a commercially available pressure-sensitive tape is manually scraped off and dissolved in chloroform. This solution is used to cover a small part of the lower surface of tipless cantilevers, onto which drops are to be attached. This process is performed using a stereoscopic microscope (Zeiss, Germany) with the aid of a thin metal wire or with an optical fiber. Special care has to be taken not to deposit any glue on the reflective surface of the cantilever. It is convenient to use mouth and head covers to avoid any contamination of the mercury surface drop. In this way, mercury drops are firmly attached and pinned to the cantilevers. A mercury drop pinned to a tipless cantilever using this procedure is shown in Figure 4.

**Measurement of the radius of curvature of standard Si_3_N_4_ tips:** As mentioned in the introduction, we first measure the adhesion force between standard Si_3_N_4_ tips in contact with freshly cleaved mica to validate our general method. The radius of these tips was obtained using the tip–shape deconvolution method [28–29] on a test grating (TGT1 from NDT-MDT Co., Russia) intended for 3D visualization of scanning tips (Figure 1). The grating with the sharp peaks was inspected with standard Si_3_N_4_ SPM cantilevers in contact mode. The scans were obtained with a scanning probe microscope in vacuum (1 × 10^−4^ Pa). The inverted images of the tip (not presented in this work) represent the sum of the experimental cantilever tip radius-of-curvature and the sharpened grating sharp peak.

**AFM and force–displacement curves:** Force–displacement curves were obtained with a scanning probe microscope (JSTM-4200 JEOL, Japan) with an 80 × 80 μm scanner that has an integrated chamber to work under vacuum (better than 1 × 10^−4^ Pa) or in an inert atmosphere. Vacuum evacuation is performed with a 300 L/s magnetic turbo molecular pump and a rotatory pump, both properly isolated from the AFM head to avoid spurious vibrations.

**Figure 5:**
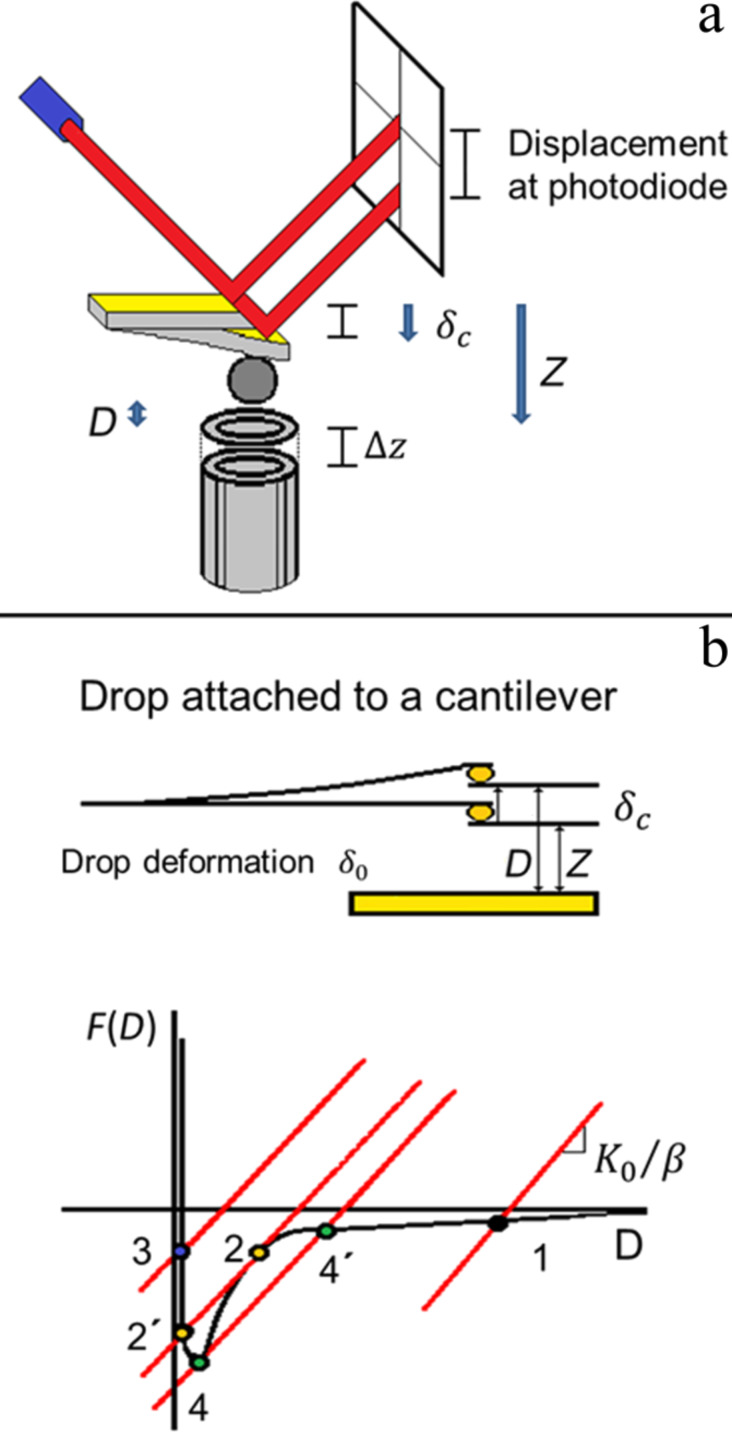
a) Schematics of the mercury drop–surface system in an AFM. *D* is the actual distance between mercury drop and surface, whereas *Z* is the distance between the drop and the cantilever at rest position and the surface that is ramped along the vertical position by the piezo. δ_c_ is the cantilever deflection and Δ*Z* is the displacement of the piezo. In general, *Z* and *D* differ due to the cantilever and drop deformations. b) Graphical construction of an AFM force–displacement curve. Lines represent the effective force (cantilever + drop deformation). Colored dots are mechanical equilibrium points given by the intersection of the lines and the surface drop interaction *F*(*D*): 1, 2, 3, 2′, and 4′. When the force gradient is larger than the effective elastic constant, the cantilever becomes unstable generating two discontinuities from which hysteresis follows: The jump-to-contact (2–2′) in the approach curve and the jump-off-contact in the withdrawal curve (4–4′).

Hooke's law gives the tip–sample force, *F*_c_ *=* −*k*_c_δ_c_, while the drop deformation will also be considered to be elastic, *F*_d_ = −*k*_d_δ_d_ [10], which is a consequence of the high surface tension of mercury (ca. 486.5 mN/m); *k*_c_ and *k*_d_ are the force constants, and δ_d_ and δ_c_ correspond to the deformation of cantilever and drop, respectively. These relevant distances are related by *D* = *Z* − (δ_c_ *+* δ_d_), where *D* is the actual distance between mercury drop and surface (Figure 5). We neglect the sample deformation because it is much smaller than the deformation of the liquid. We follow the standard theoretical development to interpret force–displacement curves as presented in [30]. The force–displacement curve is the result of the probe–sample interaction *F*_ps_(*D*), and of the elastic forces due to the deformation of both the cantilever and the drop. The former is the outcome of the addition of interactions among all atoms in the surface and the probe, which are of the type ***F***_at_ = −**A**/*D*^7^ + **B**/*D*^13^, where **A** and **B** are constants. This leads to a complex force expression between actual probe and surface. The cantilever–surface interaction is described by means of three potentials: *U*_tot_ = *U*_ps_(*D*) + *U*_c_(δ_c_) + *U*_d_(δ_d_), i.e., the potential between probe and sample, the elastic potential of the cantilever, and the potential that describes the drop deformation, respectively. The relation between δ_d_, and *Z* can be determined from the measured value of δ_c_ as a function of the elastic constants, when the system is forced to be stationary:


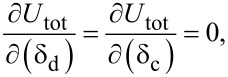


and the probe–sample force is assumed to be


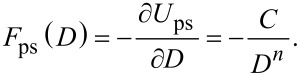


We just include here the attractive part of *F*_at_ since the repulsive interaction does not play a role in the following stability analysis. The condition for the mechanical system to be in stable equilibrium during the approach and retraction is


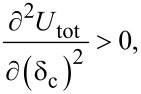


which leads to


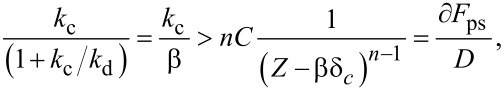


where *k*_c_/β is referred to as the effective elastic constant, and β = (1 + *k*_c_ + *k*_d_). Therefore, if the force gradient is larger than the effective elastic constant, the cantilever becomes unstable and jumps onto the surface during the approach generating the jump-to-contact discontinuity (2–2′ in Figure 5b). During the retraction, the tip follows a different trajectory than during the approach (4–4′ in Figure 5b) giving rise to force–displacement curve hysteresis. The two discontinuities in the force values are called jump-to-contact in the approach curve and jump-off-contact in the withdrawal curve. It is important to mention that this stability analysis is a simplification of a very complex process happening during the release from contact. In particular, it does not take into account the fact that this is a fracture mechanics problem where stress concentrates at the crack edge.

**Cantilever force constant:** To calculate the force constant of the cantilever we use the thermal noise method that appeals to the energy equipartition theorem, which states that the thermal energy contribution of each quadratic term in the Hamiltonian of a system is *k*_B_*T*/2 where *k*_B_ is the Boltzmann constant and *T* is the absolute temperature. The cantilever is modeled as an ideal spring with a spring constant *k*_c_. The thermal noise of the mean square displacement, 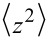, allows us to determine the constant using 
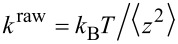
 [31–32]. With a spectral analyzer (Stanford Research Systems, 760 FFT, USA), we identify the peak corresponding to the first fundamental resonant mode of the cantilever, and a Lorentzian curve is fit to that peak to obtain the power spectral density (PSD) of the fluctuating cantilever. The area under the peak gives the 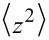 raw data. With the cantilever, a plot of the force curve vs position is determined. From this plot, we measure the slope of the contact part of the force curve; the inverse slope is the deflection calibration factor, *s*. We obtain a raw spring constant *k*^raw^ using the calibration factor *s*. This *k*^raw^ has to be corrected, because when the optical beam deflection technique is used, the inclination at the end of the cantilever is measured instead of the deflection itself, i.e., *z*(*L*) = (2*L*/3)d*z*(*L*)/d*x*, where *L* is the cantilever length. Then, 
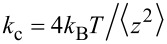
 [32]. The *k*_c_ we measure with this technique is in fair agreement with the nominal value given by the manufacturer. In most of our determinations, *k*_c_ was measured before and after each experiment to assure the cantilever integrity. If a significant change was noted the cantilever was discarded.

### Validation of the measuring procedure: Force of adhesion between standard Si_3_N_4_ tips and flat surfaces

**Figure 6:**
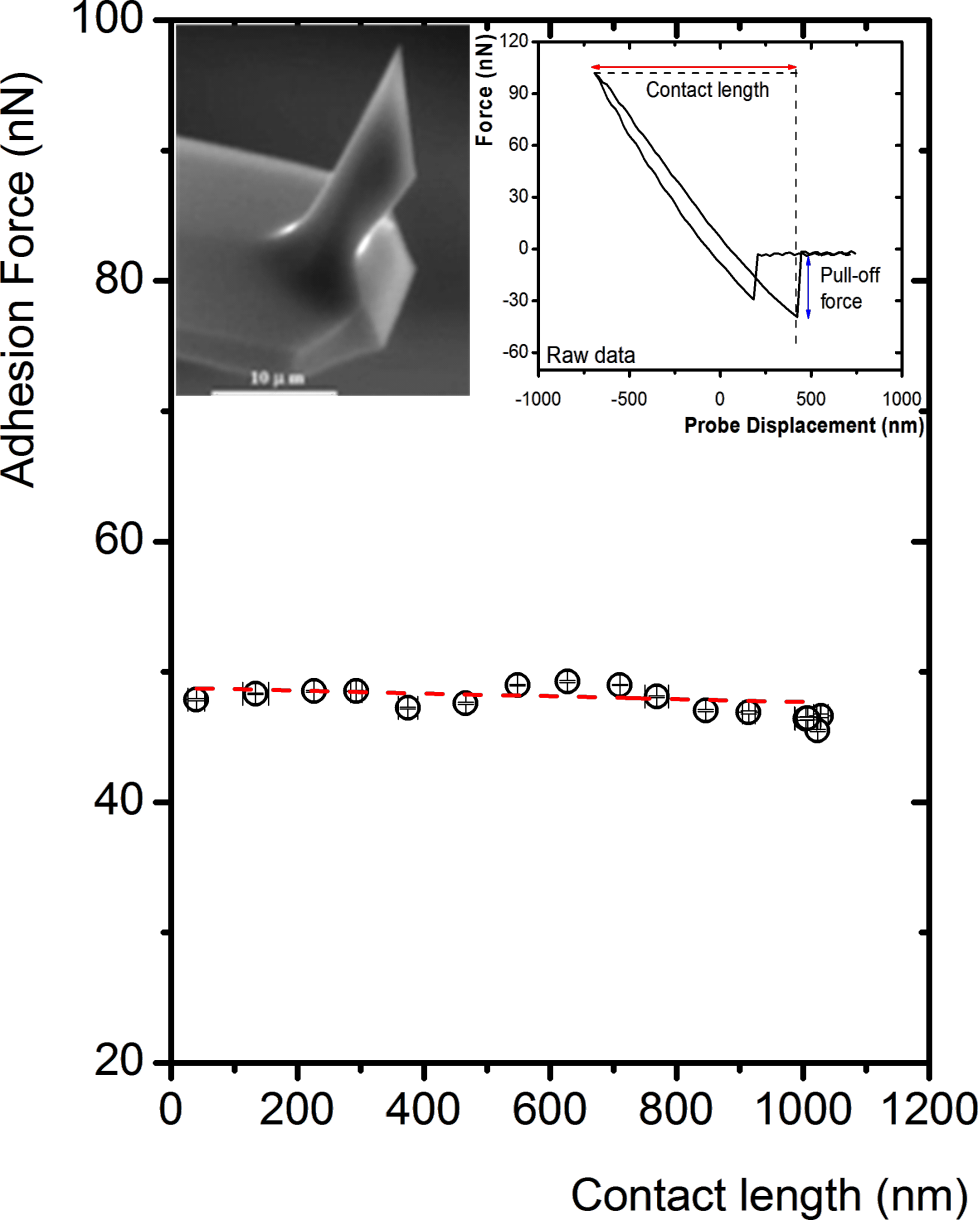
Pull-off force vs contact length; the red dash straight line is a guide to the eye. Right inset: A typical force-displacement curve on mica measured with a Si_3_N_4_ tip in vacuum. Left Inset: Typical image of an AFM Si_3_N_4_ tip obtained with a JSM5600-LVmicroscope*.*

**Mica–standard Si_3_N_4_ tip:** To check and validate our method for determining the pull-off force, *F*_adh_, we first measure the force of adhesion between a Si_3_N_4_ commercial AFM tip (CSC17, μmasch, Estonia) and a freshly cleaved Muscovite mica (S&J Trading Inc., USA) surface in vacuum (1 × 10^−4^ Pa). The inset of Figure 6 presents a typical force–displacement curve. Here, we determine *k*_c_ = 0.141 N/m, which is close to the nominal value (0.18 N/m) given by the manufacturer. The measured radius of curvature of the tip is 46.9 *±* 1.9 nm. This is larger than the nominal value provided by the manufacturer (ca. 8 nm). Force of adhesion measurements were done five times in four different areas of the sample and resulted in an average pull-off force of *F*_adh_ = 45.9 ± 0.9 nN. Figure 6 presents *F*_adh_ as a function of the difference between the maximum height reached by the piezo and the height at the jump-off-contact, which will be called hereafter “the contact length”. This quantity is a measure of how much the tip is in contact with the surface due to the compression exerted by the cantilever during a measurement. As expected for the contact between stiff solids, the pull-off force measured with a tip is nearly independent of the contact length. We also measure *F*_adh_ in air, under ambient conditions, to be able to compare the value with published data. In this case, we obtain *F*_adh_ = 48.5 ± 0.1 nN (*k*_c_ = 0.346 N/m). The measured *F*_adh_ in air is close to the value measured in vacuum, revealing the low humidity of our ambient conditions because forces of capillary origin seem to be negligible. Leite et al. [33] measured *F*_adh_ = 26.6 ± 0.4 nN in air at a relative humidity of (46 ± 3)% (*k*_c_ = 0.11 ± 0.02 N/m) with a tip curvature radius of 30 ± 5 nm. Eastman et al. [34] measured *F*_adh_ = 51 nN with commercial tips and Lomboy et al. [28] measured 50.7 ± 0.7 nN in air with a tip curvature radius of 35 nm. Because mica is a natural product the samples of which are not strictly the same, plus considering the contribution of humidity of other studies, we consider our results reasonably and close to those previously measured in air, under ambient conditions.

**Multi-scaled rough diamond surface–Si_3_N_4_ tip:** Using the same procedure as in the case of mica, we measure *F*_adh_ in vacuum between the Si_3_N_4_ tip and the multi-scaled rough diamond surface described in the Experimental section. The average for 20 measurements along five different areas is *F*_adh_ = 9.0 ± 1.0 nN. We attribute the significant relative deviation from the mean to the high rugosity of the sample. In addition, to confirm that the force of adhesion in these surfaces depends inversely on the roughness, we measure *F*_adh_ on two less rough diamond surfaces with similar chemical composition. Even though their chemical compositions are not identical, the differences in roughness are comparatively larger and are enough to explain the wetting differences with mercury as characterized by contact angle measurements [26]. One surface is the original boron-doped microcrystalline diamond film previous to the oxidation described above, with small rugosity and *F*_adh_ = 24.0 ± 1.2 nN. The other is a boron-doped polished single-crystal natural-type diamond surface with *F*_adh_ *=* 40.7 ± 1.5 nN. Both, the larger *F*_adh_ and the smaller relative measurement error correlate with the smoother quality of the corresponding surfaces.

## Results and Discussion

Here, we present pull-off force experiments on three different systems: a) an array of sharp silicon peaks and both a liquid probe and b) a hollow glass colloidal probe –the experiments with this colloidal probe allow us to contrast the results using a liquid or a solid probe– and, finally, c) a hydrophobic multi-scaled rough diamond surface and a liquid probe. It is important to mention that while we report pull-off forces, we do not do the same for the pull-on forces in these experiments. These pull-on forces are much smaller than the pull-off forces, probably because the force gradient is larger than the effective elastic constant. As a consequence, the measured forces are different during the approach and withdrawal, as explained in the discussion of Figure 5.

### Force of adhesion between both a solid spherical and a liquid probe, and different surfaces

The following experiments were performed in a dry N_2_ atmosphere to avoid capillary forces due to water condensation and, in the case of the mercury drop, also to prevent oxidation. Measurements were carried out in many different areas along the surfaces, with 15–20 measurements per area. At these small scales, it is not uncommon that during the scanning using hollow glass spheres or liquid probes, we find defects or adhered particles on the surface, e.g., broken peaks, holes, powder, surface debris, which are not visible with optical microscopy. Therefore, the highest and lowest force values in each area were not considered. Moreover, we did also not consider a few areas whose results presented a significant large standard deviation.

**Figure 7:**
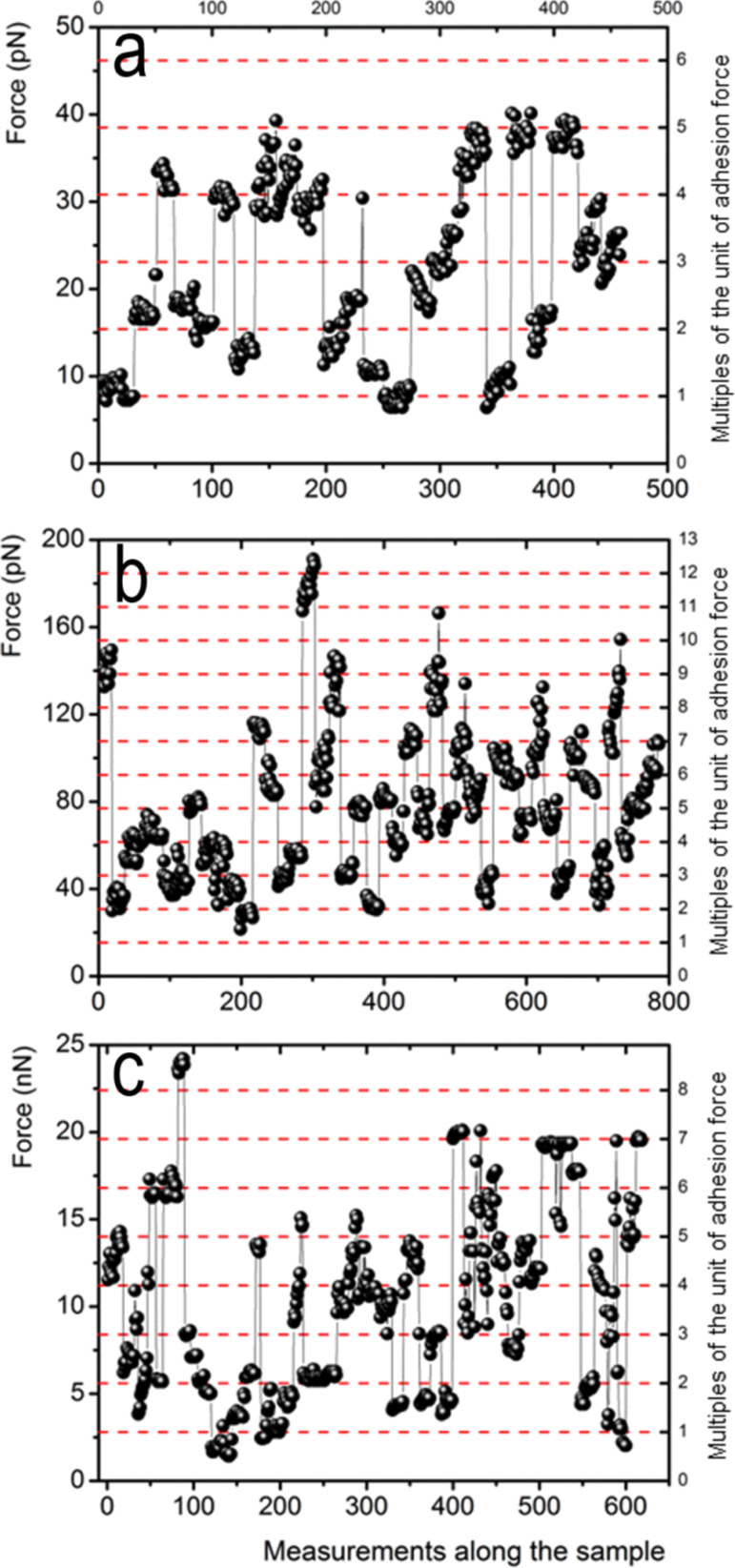
Results for pull-off force measurements carried out along different surfaces. The pull-off force between the array of sharp silicon peaks and both (a) a hollow glass sphere, and (b) a mercury drop. (c) The pull-off force between the multi-scaled rough diamond surface and a mercury drop. Measurements were carried out on 26, 44, and 51 different areas of the surfaces, respectively. Right axes and red dashed lines are the same pull-off forces plotted on the left axes, but divided by the unit force of adhesion that is different for each system, as explained in the text.

**Array of sharp silicon peaks–hollow glass probe:** We did experiments with a colloidal probe to check the validity of the method used to determine the pull-off force with the liquid sphere, and also to compare the results from colloidal and liquid probes. We measured *F*_adh_ between the array of sharp silicon peaks (Figure 1) and a hollow glass sphere (*r* = 33.8 μm, 3M USA) attached to a tipless cantilever (*k*_c_ = 2.35 × 10^−4^ N/m). The force constant is small because we selected a relatively large tipless cantilever. Measurements were carried out in 26 different areas along the grating resulting in 459 measurements, which are presented in Figure 7a. The standard deviation of F*_adh_* was determined for each measured area. The average of these standard deviations is 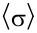 = 1.44 pN.

**Array of sharp silicon peaks–mercury drop:** *F*_adh_ between the array of sharp silicon peaks and a mercury drop attached to a tipless cantilever (*k*_c_ = 5.89 × 10^−3^ N/m) was measured. Measurements were carried out on 44 different areas along the grating, resulting in 800 measurements, which are presented in Figure 7b for a drop radius of *r* = 14.1 μm. The standard deviation of *F*_adh_ was determined for each measured area; on the average 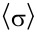 = 5.1 pN.

**Multi-scaled rough diamond surface–mercury drop:** The adhesion force between the multi-scaled rough diamond surface and a mercury drop attached to a tipless cantilever (*k*_c_ = 0.0444 N/m) was measured, following the method described above. The measurements were carried out on 51 different areas of the sample resulting in 618 measurements that are presented in Figure 7c for a drop radius of *r* = 6.6 μm. The standard deviation of *F*_adh_ was determined for each measured area; on the average 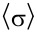 = 0.97 nN.

**Figure 8:**
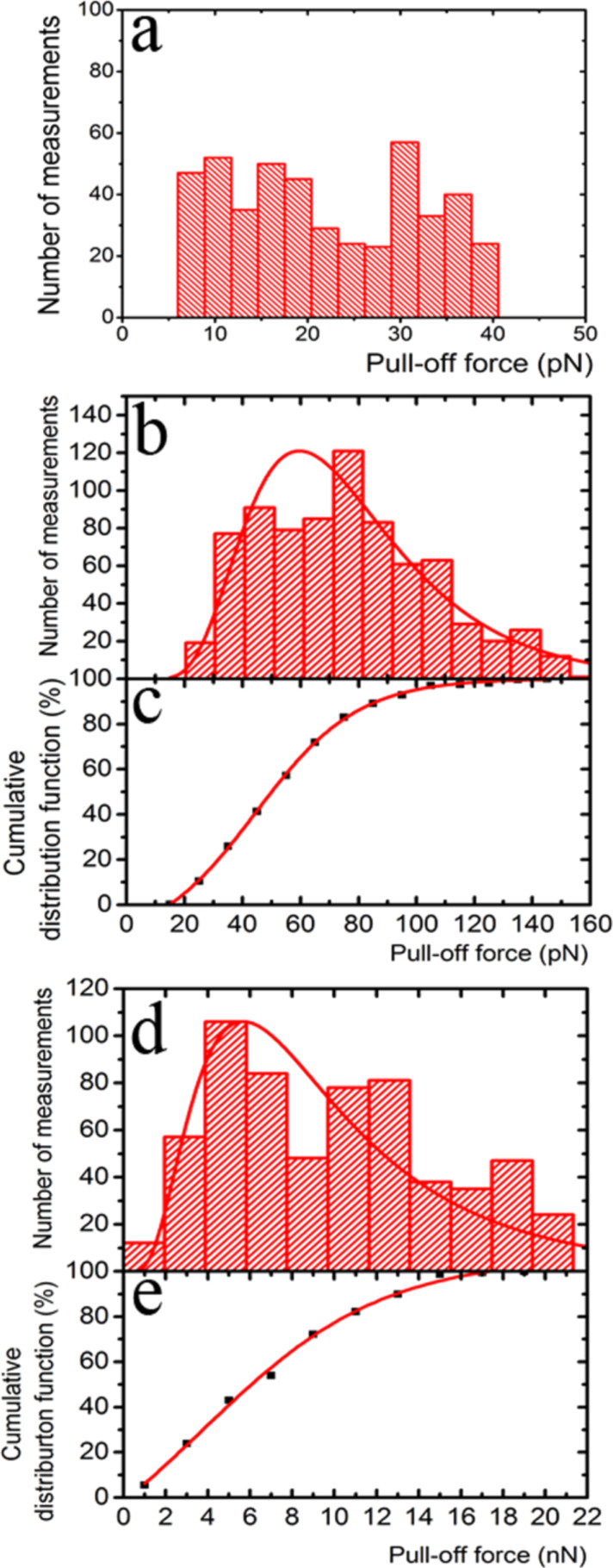
Statistics of the whole set of pull-off force measurements. Distribution of the pull-off force between an array of sharp silicon peaks and: (a) a hollow glass sphere; (b) a mercury drop; (c) cumulative frequency chart for the latter; (d) distribution of the pull-off force between the multi-scaled rough diamond surface and a mercury drop, and (d) its corresponding cumulative frequency chart. Bin size = 2σ for all distributions. In the distribution charts (b) and (d) the red line is a log-normal fit (a guide to the eye). In the cumulative frequency charts, the red line corresponds to a Boltzmann sigmoidal fit that is also a guide to the eye.

In Figure 7, we observe the same pattern for all three cases, the data form clusters that correspond to measurements in one particular area, i.e., data dispersion for a given area is relatively small. However, when all the data for each case are compared, their dispersion is high without any apparent reason. The measured values span forces from 5 to 40 pN, from 20 to 190 pN, and from 2 to 25 nN. We first describe the statistics of the obtained results as follows: We construct distributions of occurrences of the pull-off force for each system; a bin size of 2σ was selected for each case, where σ is the average standard deviation for every sample tested. In Figure 8, we present the force distributions for each case. For the system composed of the array of sharp silicon peaks and the hollow glass sphere (Figure 8a), the distribution is relatively flat with a mean value of the pull-off force of ca. 22.0 pN. In the case of the pull-off force between the array of sharp silicon peaks and a mercury drop (Figure 8b), the distribution is unimodal. A fit of the data to a log-normal curve that is an aid to the eye presents a maximum at about 60.02 pN. The unimodal distribution is a feature not observed in our colloidal-probe measurements. Finally, Figure 8d presents the distribution for the system composed of the multi-scaled rough diamond surface and the mercury drop. A log-normal fit to the data is also included as a guide to the eye and shows a maximum at 5.7 nN. Figure 8c and Figure 8e presents cumulative frequency charts for the pull-off force data for the rough surfaces and the mercury drop. It is important to note that because of the low conductivity of this surface, the polarization of the drop surface due to contact electrification could play a role after several approaches in successive measurements. Therefore, during some experiments, we included a small radioactive source (^241^Am, LEA-CERCA, France) close to the cantilever to get rid of charges. No effect was observed, so we consider this effect to be negligible.

**Figure 9:**
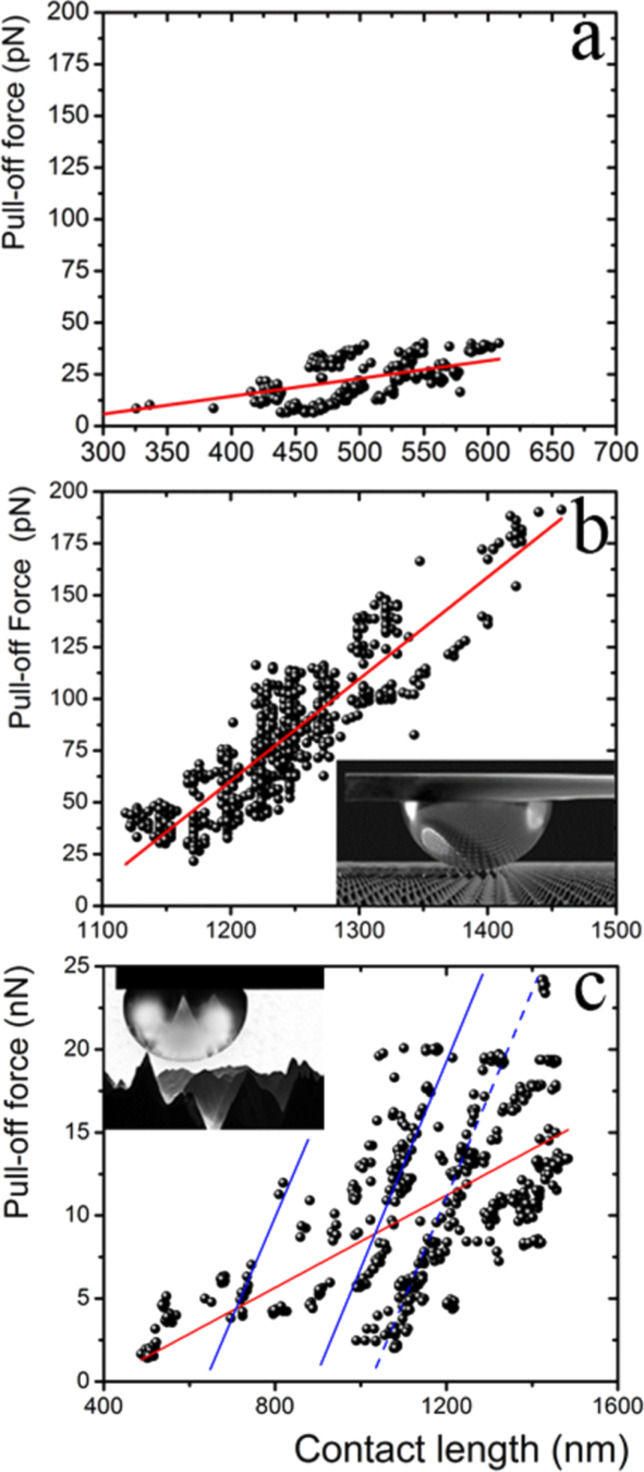
Adhesion force vs contact length between the array of sharp silicon peaks and (a) a hollow glass sphere and (b) a mercury drop; and (c) between the multi-scaled rough diamond surface and a mercury drop. Measurements are performed on different areas of the studied surfaces. The red line corresponds to a linear fit. Inset in (b): Sketch of a mercury drop attached to a tipless cantilever in contact with the array of sharp silicon peaks. Inset in (c): Sketch of a mercury drop attached to a tipless cantilever in contact with the sub-micrometer protrusions of the multi-scaled rough diamond surface.

To help to build a physical interpretation of the experimental results, we plot in Figure 9 *F*_adh_ as a function of the contact length, which, as mentioned above, measures to some extent how much compression is made on either the liquid drops or on the solid tips attached to the AFM during a measurement. The pull-off force measured between the array of sharp silicon peaks and the hollow glass sphere (Figure 9a) is not as insensitive to the contact length as in the case of a tip and a flat surface (Figure 6). This result probably reveals some compression of the silicon peaks taking place, or/and a small arrangement of the sphere during the compression after making contact with the sharp silicon peaks of the grating since they are not exactly of the same height. As mentioned above, the actual height of these peaks ranges from 0.3 to 0.7 μm. When a softer probe, such as the mercury drop, is employed, in addition to the compression of the silicon peaks or/and small probe arrangements due to the height dispersion, the compression of the drop needs to be taken into account too. Figure 9b presents the results for the mercury-drop probe (inset of Figure 9b) as a function of the contact length, which are equivalent to those of Figure 9a. Here, we clearly observe that the pull-off force correlates positively with the contact length. This result seems reasonable because the compliance of the drop, although small, allows for making contact with more sharp peaks of the grating for larger compressions. Figure 9c presents *F*_adh_ as a function of the contact length for the multi-scaled rough diamond surface. As above, here we also observe that the pull-off force correlates positively with the contact length but in a more complex way. The red line depicts a linear fit of all data. Here, the correlation is notably lower than in the case of the array of sharp silicon peaks. The data seems to be split into groups apparently aligned (blue lines). We consider that this dispersion agrees with the fact that a liquid probe will interact differently with each particular area under measurement along the sample because the surface is not only very rugose but also highly heterogeneous, with peaks of widely varying heights, shapes and relative distances (Figure 2). In summary, larger contact lengths lead to larger deformations of the solid or liquid probe, and possibly of the substrate, which results in a larger pull-off force due to the increase of contacts. We point out that the pull-off forces for the interaction of the array of sharp silicon peaks with both glass sphere and mercury drop are of the same order of magnitude. In contrast, the adhesion force between the drop with the multi-scaled rough diamond surface is three orders of magnitude larger.

**Figure 10:**
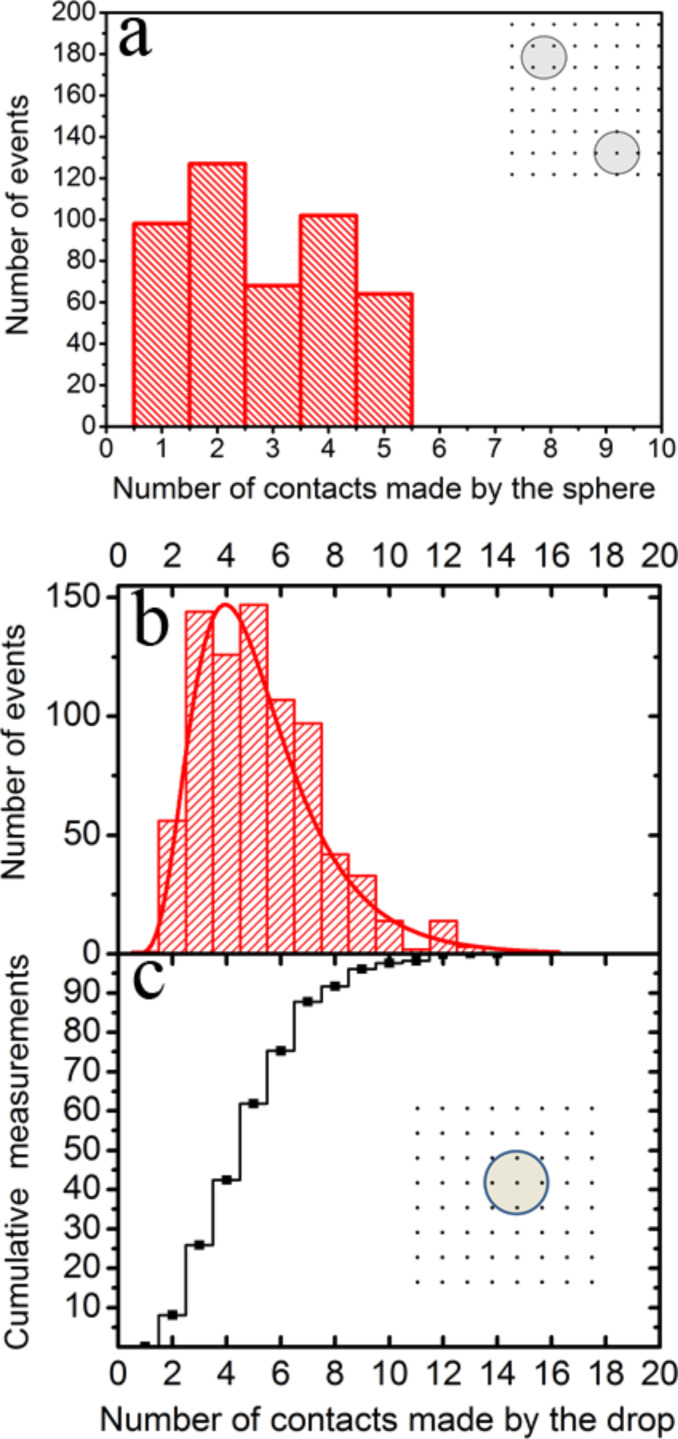
Distribution of the number of contacts with the array of sharp peaks: a) The number of contacts made with the glass sphere is between 1 and 5. b) The number of statistically significant contacts with the mercury drop is between 2 and 9. The red line is a log-normal fit as a guide to the eye. c) Cumulative frequency chart of the number of peaks of the array that interact with the mercury drop (b). The insets in (a) and (b) present sketches of the possible ways in which the lower parts of the sphere or the drop, as observed from above, can interact with the peaks.

Our interpretation of the results with the glass sphere is as follows: The glass may touch quite a few sharp peaks of the grating at the jump-to-contact. However, after the cantilever has compressed the sphere, it may now be in contact with more peaks than before. Therefore, the measured pull-off force must be, to a first approximation, a multiple integer of the force corresponding to the interaction of a single peak with the sphere at the jump-off-contact. Therefore, this unit of force can be numerically estimated from the pull-off force data as follows. Starting with a test number for each system, close to the lowest data values of *F*_adh_, we divide all the pull-off force data by it. The resulting values are gathered inside a set, Set 1. These numbers happen to be statistically close to integers; so they are approximated to the nearest integer. These integers are gathered inside a second set, Set 2. We then choose the unit of force or force per peak as the test number that yields the best correlation regression coefficient (*r* = 0.983) between both sets of numbers. Following this procedure, we obtain *F*_adh_/peak = 7.7 pN for the system composed of sharp peaks and glass sphere. The integers obtained in this way are an estimate of the number of contacts between the glass sphere and the peaks for each run. The number of contacts we find in this system ranges between 1 and 5 as shown in Figure 10a. The low number of contacts in this case, the relative insensibility of the pull-off force to the contact length, and the high stiffness of the glass sphere could explain why the distribution of the pull-off force between the array of sharp silicon peaks and the hollow glass sphere is relatively flat (Figure 8a). We follow the same reasoning to interpret the results of the force of adhesion measurements with the liquid probe. In this case, we obtain *F*_adh_/peak = 14.9 pN (*r* = 0.992). This value is larger than the *F*_adh_/peak obtained for the glass sphere because wetting is probably playing a significant role; the grating is not supersolvophobic for mercury. Interestingly, this number is close to the smallest values of the pinning forces measured for a contact line pinned on a strong defect on a carbon nanotube dipped in a liquid, also measured by AFM [35]. Just as before, dividing each measured pull-off force by 14.9 pN, we obtain numbers that are statistically close to integers. In this way, we get the approximate number of contacts between the drop and the array of peaks in a measurement. These results are presented in Figure 10b and Figure 10c. It is important to keep in mind that both the solid and liquid spherical probes can interact with more peaks than ideally expected (between 1 and 4) because the peaks of the grid have a significant height dispersion.

**Figure 11:**
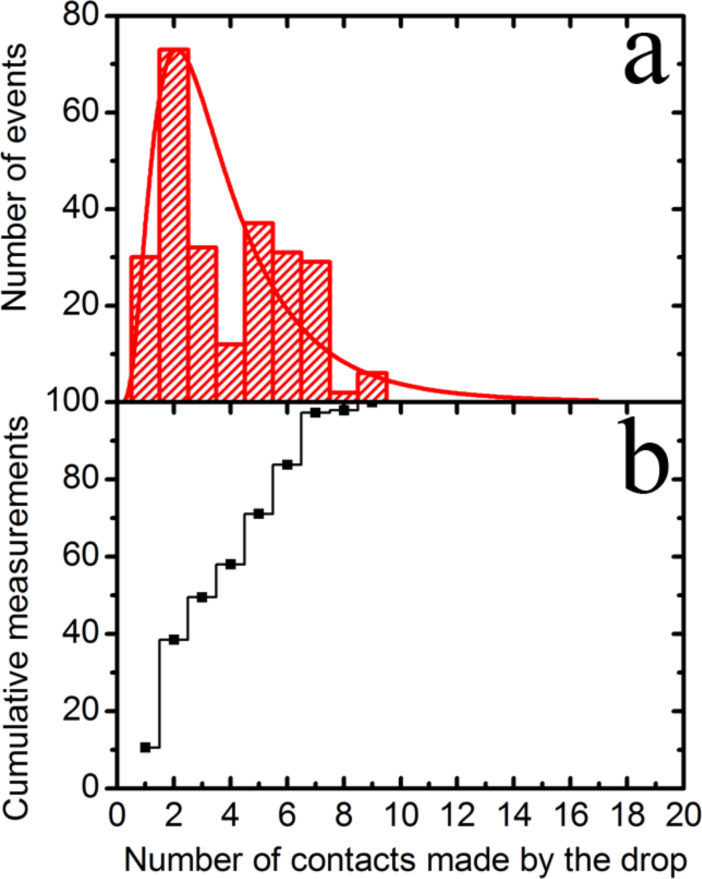
a) Distribution of the number of contacts of the multi-scaled rough diamond surface with the mercury drop for the group with the most data points aligned along a straight line in Figure 9c (dashed line). The red line is a log-normal fit as a guide to the eye, b) Cumulative frequency chart of the number of contacts that interacts with the protrusions of the diamond surface.

For the case of the multi-scaled rough diamond surface in contact with the mercury drop, we repeat the numerical analysis performed above to gain some understanding about the jump-off-contact process. However, due to the considerable data dispersion, in this case, we made the analysis just for the sub-group with the most data points aligned along a straight line in Figure 9c (dashed line). Once again, the unit force corresponds to the interaction between a single sub-micrometer protrusion and the drop, and in this case it is equal to *F*_adh_/protrusion = 2.8 nN (*r* = 0.994). This value is of the same order of magnitude as the average taken over several areas made with an AFM Si_3_N_4_ tip (*F*_adh_ = 9.01 nN) determined above. The corresponding distribution for contacts is given in Figure 11 along with its cumulative chart. According to this analysis, the drop can make contact with 1–7 protrusions of the diamond surface, with a maximum likelihood found for contact with two protrusions. Despite the rougher quality of the multi-scaled diamond surface, the number of peaks touching the surface of the drop is still small.

In Figure 7, we highlight with red dashed lines the multiples of the unit of force (right axes) for each of the cases we discussed above, and how the force measurements cluster around to these lines. Also, it is important to note that in the case of the multi-scaled rough diamond surface, the value of *F*_adh_/protrusion from the sub-group with the most data points aligned along a straight line (dashed line in Figure 9c) was taken as the unit of force to rescale the whole data of Figure 7c. Apparently, this unit of force is appropriate to describe the data for the whole surface.

Above, we obtained the pull-off force between the mercury drop and both a sharp peak and a sub-micrometer protrusion of the multi-scaled rough diamond surface. Now, it would be interesting to assess if the experimental values, *F*_adh_/peak = 14.9 pN and *F*_adh_/protrusion = 2.8 nN, are reasonable. To do this, we now estimate the pull-off force between the droplet and a peak or a sub-micrometer protrusion, using a very simple model as a first-order approximation. We assume that the forces acting upon a drop at the moment of the jump-off-contact are two elastic restoring forces and one attractive force due to adhesion. The latter would be considered as binary, a non-zero value before the pull-off and zero after it. The elastic forces correspond to the cantilever (*F*_c_ = –*k*_c_δ_c_) and to the surface drop deformation (*F*_d_ = –*k*_d_δ_d_) acting along the vertical direction. As a consequence, they will behave like a spring with an effective constant 
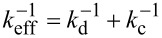
.

According to these assumptions, the energy of the whole system can be estimated by


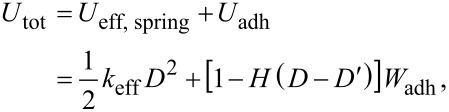


where *H*(*x*) is the Heaviside function, D′ is the distance just when the jump-off-contact occurs, and *W*_adh_ is the energy of adhesion between the contacting surfaces at the moment of jump-off-contact. For stationary motion, the following relationship is valid:


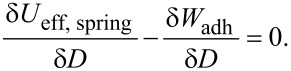


Integrating this equation, we obtain that just at the jump-off-contact, (1/2)·*k*_eff_D′^2^ = *W*_adh_. Then, D′ can be calculated as


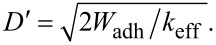


On the other hand, at the jump-off-contact, the force is also given by the effective spring constant, *F*_jump-off-contact_ = *k*_eff_·D′. Therefore, if *W*_adh_ is known, *F*_jump-off-contact_ can be evaluated through *D*′.

First, we discuss the case of the pull-off force between the drop and the multi-scaled rough diamond surface. The work of adhesion, i.e., the energy of adhesion per unit area, *w* = *W*_adh_/unit area can be obtained with the Dupré equation for wetting *w* = γ(1 + cos θ_c_). Using the contact angle between mercury and a boron-doped single crystal natural-type diamond with a polished surface, θ_c_ = 155° [10], we obtain *w* = 0.0454 J/m^2^. To exactly determine the area of the rough diamond surface that is wetted by mercury is a difficult task because the sub-micrometer protrusions are not uniform and they are randomly distributed [26]. However, we can estimate this value from the topographic amplification of an AFM image (Figure 2d) of a typical sub-micrometer protrusion. To do this, we evaluate the average height of the protrusion as a function of the radial distance measured from its center (Figure 3). We estimate that the area of contact with the drop of a typical protrusion is approximately a spherical cap, with a radius of 30 nm and a height of 5 nm. This results in a value of *W*_adh_ = 132.3 × 10^−18^ J. Now to evaluate *k*_eff_, the spring constant of the mercury surface is needed and it can be estimated from experiments of cylindrical nanofibers dipped in liquids of different γ, where γ is the liquid–vapor surface tension [36]. For contact angles above 50–60°, the spring constants are almost insensible to the contact angle, and although these authors did not explore angles corresponding to hydrophobic surfaces (θ_c_ > 90^o^), they estimated that *k*_d_ ≈ 0.52γ for a large ratio between the lateral size of the meniscus and the radius of the fiber.

With these numbers, we obtain *k*_eff_ = 37.6 × 10^−3^ N/m, i.e., the cantilever is the dominant spring, and *D*′ is calculated. After all these approximations are made, the force of adhesion between the mercury drop and a sub-micrometer protrusion is estimated to be *F*_jump-off-contact_ ≈ 1.42 nN. This value is very close to our experimental value *F*_adh_/protrusion = 2.8 nN*.*

We repeat similar calculations for the pull-off force between a sharp peak in contact with the liquid probe. Nevertheless, in this case, we do not know the actual chemical composition of the sharp peaks to estimate the energy of adhesion with mercury drops. Thus, we do not know their wetting properties and the real contact area between these peaks and the drop. From the manufacturer information, the peaks are made of silicon and inevitably oxidized, with a tip radius of 10 nm. Keeping these caveats in mind, we use the Hamaker summation method to evaluate *W*_adh_ of the sharp peak-drop system as the energy between a sphere of *R*_1_ = 10 nm (sharp peak) and one of *R*_2_ = 14.1 μm (Hg drop) separated by a distance *D* = 0.5 nm, i.e., atomic contact*,* using *W*_adh_ = −*AR*_1_*R*_2_/6*D*(*R*_1_ + *R*_s_) [37]. Here, *A* is the Hamaker constant that can be evaluated with the same method as the interaction energy between two flat surfaces of unit area, at the same separation *D*, using the Dupré equation *w* = *A*/12π*D*^2^ = γ(1 + cos θ_c_).

For this calculation, we use θ_c_ = 137° as the contact angle between mercury and a silicon wafer [38], which results in *w* = 0.130 J/m^2^. Since *k*_eff_ = 5.7 × 10^−3^ N/m (here the cantilever is also the dominant spring) and after calculating *D*′ as mentioned above, the result of the peak–mercury drop force of adhesion is ca. 216 pN. Although this estimation is an order of magnitude larger than the obtained results for the sharp peak–drop force AFM measurements, given the many approximations used for this evaluation, we consider our results to be reasonable.

## Conclusion

A method was developed to perform and interpret pull-off force measurements during the jump-off-contact process between a micrometric liquid drop attached to an AFM tipless cantilever and rough surfaces. The measurements were made with an atomic force microscope in nitrogen atmosphere.

Remarkably, the data naturally cluster around integer multiples of a unit of interaction force between the macroscopic drop and the surface that corresponds to the interaction with a single peak or protrusion. This unit of force is of the order of a few piconewtons for the case of the solid sphere and peaks of nanometer radius. These values are of the same order of magnitude as the smallest ones found for the contact between nanotubes and liquids [35]. It could be possible that the unit of force that we measure corresponds to a single pinning point of the contact line. This suggests that the method we have reported here can give useful information about fundamental wetting properties of rough materials. On the other hand, for larger protrusions of the order of tens of nanometers where macroscopic contact angles hold, this method provides values of the force of adhesion that could be useful for the general characterization of the interaction between liquids and rough surfaces. It is also important to note that the multiple subgroups issue found in the pull-off force vs contact length for the case of the liquid drop (Figure 9c) needs further investigation because multiple subgroups do not appear in the contact of a liquid drop and a well-ordered rough surface. The different scales of the multi-scaled rough surface surely are playing an important role. This work is underway.
